# A Solid-in-Oil Nanodispersion System for Transcutaneous Immunotherapy of Cow’s Milk Allergies

**DOI:** 10.3390/pharmaceutics12030205

**Published:** 2020-02-27

**Authors:** Momoko Kitaoka, Wei Xiao, Qingliang Kong, Yoshiro Tahara, Noriho Kamiya, Masahiro Goto

**Affiliations:** 1 Department of Applied Chemistry, Graduate School of Engineering, Kyushu University, Fukuoka 819-0395, Japan; kitaoka.momoko.032@m.kyushu-u.ac.jp (M.K.); xiaowei939413@hotmail.com (W.X.); kong.qingliang.445@s.kyushu-u.ac.jp (Q.K.); ytahara@mail.doshisha.ac.jp (Y.T.); kamiya.noriho.367@m.kyushu-u.ac.jp (N.K.); 2 Advanced Transdermal Drug Delivery System Center, Kyushu University, Fukuoka 819-0395, Japan; 3 Center for Future Chemistry, Kyushu University, Fukuoka 819-0395, Japan

**Keywords:** cow’s milk allergy, whey, solid-in-oil nanodispersions, transcutaneous immunotherapy, vaccine, nanocarrier

## Abstract

An allergy to cow’s milk proteins is the most common food allergy in infants and toddlers. Conventional oral immunotherapy for cow’s milk allergies requires hospital admission due to the risk of severe allergic reactions, including anaphylaxis. Therefore, a simpler and safer immunotherapeutic method is desirable. We examined transcutaneous immunotherapy with a solid-in-oil (S/O) system. In the S/O system, nano-sized particles of proteins are dispersed in an oil-vehicle with the assistance of nonionic surfactants. In the present study, the S/O system enhanced the skin permeation of the allergen molecule β-lactoglobulin (BLG), as compared with a control PBS solution. The patches containing BLG in the S/O nanodispersion skewed the immune response in the allergy model mice toward T helper type 1 immunity, indicating the amelioration of allergic symptoms. This effect was more pronounced when the immunomodulator resiquimod (R-848) was included in the S/O system.

## 1. Introduction

Cow’s milk allergies (CMA) are the most common food allergies in infants and young children [1], and the increasing prevalence of CMA is a global health concern [2]. Many children outgrow CMA by the early years of their childhood; however, some individuals still have an allergy to cow’s milk proteins in their adulthood. Frequently observed allergic symptoms of food allergies are gastrointestinal tract inflammation and skin reactions, such as eczema, urticaria, and atopic dermatitis [3]. Less often, respiratory and gastrointestinal symptoms are also present. The occurrence of life-threatening anaphylaxis is also a serious problem regarding many food allergies, including CMA.

As with other food allergies, oral immunotherapy (OIT) with the allergen, i.e., cow’s milk, has been examined extensively in many studies, with resulting increased dose thresholds for allergic reactions [4]; however, during OIT treatment, the allergen is delivered to the systemic circulation, which has a high risk of causing severe adverse events. In response to this concern, safer approaches to food allergy immunotherapy are being explored. Recently, epicutaneous immunotherapy (EPIT) using the Viaskin epidermal delivery system demonstrated significant inductions of tolerance to peanut allergies and CMA using food antigens in placebo-controlled double-blind clinical trials [5,6]. The Viaskin system uses occlusive patches carrying lyophilized food allergens. As the patches are occlusive, the stratum corneum (SC), a hydrophobic surface barrier in the skin, is hydrated with naturally emerging perspiration, thus allowing the allergens in the patch to dissolve in the moisture and penetrate through the SC. Using Viaskin, the risks of severe adverse events are expected to be low; however, the efficiency of the antigen delivery is also low due to the passive dispersion that occurs when using the occlusive patches. Moreover, efficiency of adjuvant delivery is low as well.

Another effective approach to enhancing the efficacy of immunotherapy is the use of immune response modulators [7]. These molecules include ligands of pattern recognition receptors, such as lipopolysaccharides, nucleic acids, lipoproteins, and peptides originally derived from bacteria or viruses and their analogues. Among them, R-848 (resiquimod), a guanine nucleoside analogue, is known to strongly activate immune cells and skew the immune response toward T helper type 1 (Th1) immunity [8]. Being moderately hydrophobic and of low molecular weight, R-848 is suitable for topical administration [9]. However, as R-848 is lyophilic, the available strategies enabling the efficient co-delivery of R-848 and a hydrophilic antigen molecule are limited.

In the present study, we applied a solid-in-oil (S/O) nanodispersion system to deliver both the hydrophilic protein molecules and the lyophilic adjuvant through the epidermis and thus utilize the abundant immune cells in the skin for immunotherapy. In the S/O nanodispersion system, nanosized particles of hydrophilic vaccines/allergens are dispersed in an oil vehicle with the aid of hydrophobic surfactants [10]. They are prepared by freeze-drying water-in-oil emulsions containing vaccines/allergens followed by dispersion of the surfactant–medicine complexes into an oil vehicle that has affinity to the SC. A patch system can be painlessly applied to the intact skin, and the isopropyl myristate (IPM) vehicle used in the S/O nanodispersion system loosens the lipid bilayer in the SC [11,12]. This facilitates drug elution into the water moisture in the skin and consequent drug permeation through the skin. A previous work reported that an S/O nanodispersion system could efficiently deliver ovalbumin, a vaccine model protein, through the skin and induce antibody-specific antibodies [10].

Here, we examined the utility of c using a combination of R-848 and the S/O nanodispersion system to alleviate the allergic responses of whey-sensitized (CMA model) mice. The allergen β-lactoglobulin (BLG; Bos d 5) was selected to treat the CMA model mice. BLG is found in whey protein and is one of the major allergens of CMA [13]. In the present work, BLG was encapsulated in the S/O nanodispersion, after which, the release efficiency and the skin permeability of BLG were examined. R-848 was added to the S/O nanodispersion system, and, following a simple patch administration of the BLG/R-848 on intact ear skin, the amelioration of skin symptoms in whey-sensitized model mice and the changes in the serum antibody levels and cytokine profiles were examined.

## 2. Materials and Methods

### 2.1. Materials

R-848 was purchased from Enzo Life Science (Farmingdale, NY, USA). Sucrose laurate (L-195) and whey protein concentrate (WPC)392 (contains 80% protein) were kindly provided by Mitsubishi-Kagaku Foods (Tokyo, Japan) and by Fonterra (Auckland, New Zealand), respectively. β-lactoglobulin was purchased from Sigma-Aldrich (St. Louis, MO, USA). Horseradish peroxidase-labelled rabbit anti-mouse IgG1 and IgG2a were purchased from Rockland Immunochemicals (Gilbertsville, PA, USA). The Yucatan micropig (YMP) skin was purchased from Charles River Laboratories Japan (Kanagawa, Japan). Fluoroscein 5-isothiocyanate (FITC) was purchased from Tokyo Chemical Industry (Tokyo, Japan).

### 2.2. Animals

Female 4-week-old C3H/HeJ mice were purchased from Kyudo (Saga, Japan) and maintained under standardized conditions on a cow’s milk-protein-free diet (CE-2, CLEA Japan; Tokyo, Japan) or other specified diet. The animal experiments were conducted in accordance with the protocol approved by the Ethics Committee for Animal Experiments, Kyushu University (A30-240-0, approved on August 1, 2019) and the Guide for the Care and Use of Laboratory Animals (Science Council of Japan).

### 2.3. Preparation of S/O Nanodispersions

**Scheme 1:**
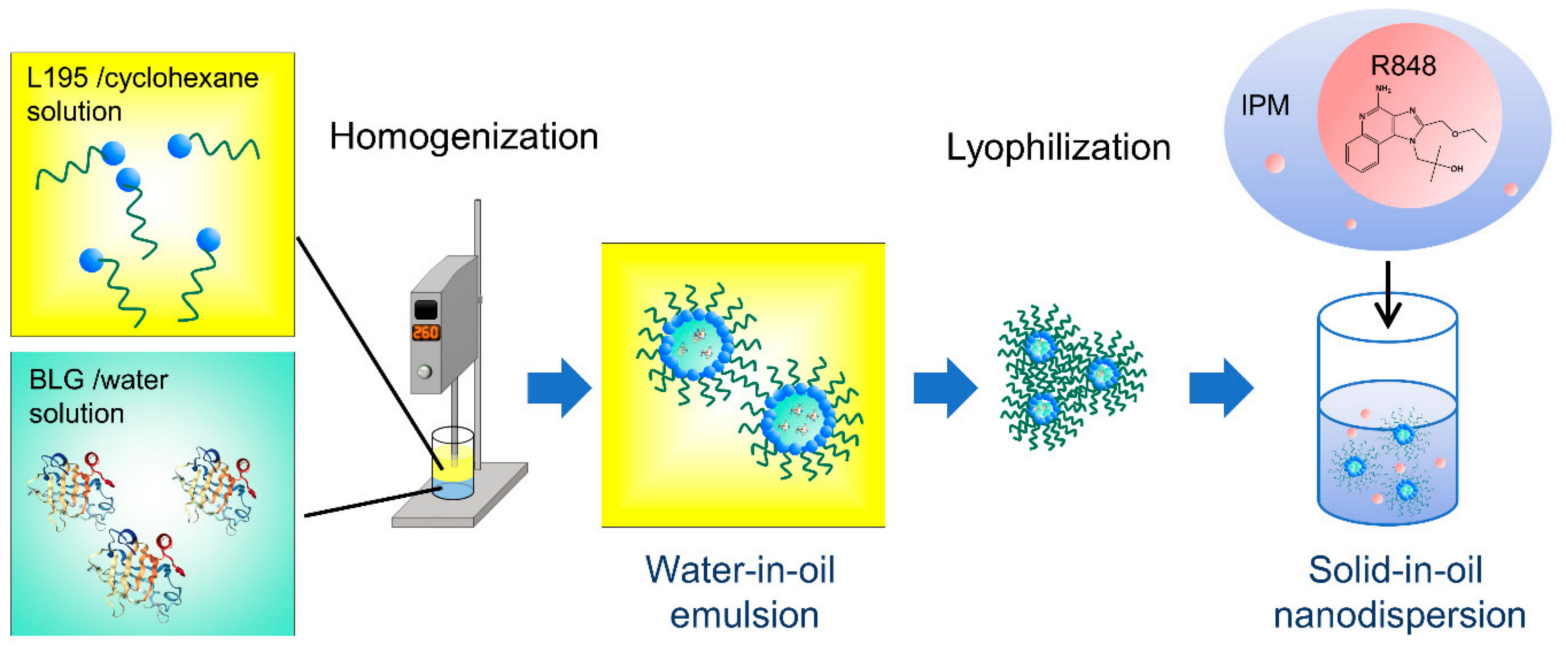
The preparation method for the solid-in-oil nanodispersion. Surfactant L195 in a cyclohexane solution and β-lactoglobulin in a Milli-Q water solution were homogenized, lyophilized, and then dispersed in an isopropyl myristate (IPM) vehicle containing the adjuvant resiquimod (R-848).

The S/O nanodispersions were prepared as previously described (Scheme 1) [14]. Briefly, an aqueous solution of BLG (1 mg/mL, 2 mL) and a cyclohexane solution of sucrose laurate L195 (25 mg/mL, 4 mL) were added to a 6 mL vial and homogenized with a polytron homogenizer (Kinematica AG; Luzern, Switzerland) at 26,000 rpm for 2 min. The obtained water-in-oil emulsion was then flash frozen in liquid nitrogen for 20 min and lyophilized until the powdery surfactant–protein complex formed. IPM containing R-848 (100 μg/mL, 2 mL) was added to the surfactant–protein complex powder and mixed with a vortex-mixer to obtain an S/O nanodispersion. The surfactant–protein complex was also dispersed in IPM in the absence of R-848 as a control.

For the preparation of the FITC-labeled BLG, solutions of BLG (5 mg/mL, 2 mL) and FITC (0.9 mg/mL, 0.2 mL) in 50 mM carbonate buffer (pH 9.0) were mixed and incubated for 2 h at room temperature in the dark. The reaction mixture was then desalted with a PD-10 column (GE Healthcare; Buckinghamshire, England) in accordance with the manufacturer’s instructions. The obtained yellow liquid was lyophilized and stored in the dark until use. The labelling ratio of BLG to FITC was 1:4 according to a calculation based on the UV absorption at pH 7.5 (Tris-HCl buffer). S/O nanodispersions of the FITC-labelled BLG were prepared as described above.

The average diameter and the polydispersity index of the particles were measured with a Zetasizer NanoZS (Malvern; Worcestershire, UK).

### 2.4. In Vitro Release Test

The FITC-BLG release efficiency from the S/O nanodispersions was examined using Franz diffusion cells (effective diffusion area: 0.785 cm^2^, receptor phase: PBS, and receptor volume: 5 mL). Nucleopore membranes (pore size φ 0.1 μm) from GE Healthcare were set between the donor and the receptor chambers. The S/O nanodispersions with or without R-848 (0.2 mL) were placed in the donor chamber and incubated for 24 h at 36 °C (*n* = 3). The receptor phase (0.2 mL) was collected at 1 h, 2 h, 4 h, and 24 h and replaced with fresh PBS. The FITC-BLG concentrations in the receptor samples were measured with a fluorescence spectrometer, LS-55 (PerkinElmer; Waltham, MA, USA).

### 2.5. Permeation of the S/O Nanodispersions into Skin

YMP skin was used as a model of human skin to examine the skin permeation of FITC-BLG applied via the S/O nanodispersion technique. Following the removal of subcutaneous fat and blood vessels, the skin was cut into 1.5 × 1.5 cm^2^ pieces and set onto Franz diffusion cells (effective diffusion area: 0.785 cm^2^, receptor phase: PBS, and receptor volume: 5 mL) [14]. The PBS solutions and S/O nanodispersions were put on the skin surfaces (0.2 mL/cell), and the cells were incubated for 24 h at 32 °C (*n* = 3). After incubation, the skins were washed thoroughly with Milli-Q water, then with 80% ethanol, and then again with Milli-Q water. The skins were subsequently cut into 16 pieces, and the FITC-BLG was extracted in PBS (1 mL) by vortexing for 18 h. The FITC-BLG concentration in the extraction solution was measured with a fluorescence spectrometer, LS-55.

The amount of FITC-BLG that had permeated through the skin was also observed using a confocal laser scanning fluorescent microscope (LSM 700, Carl Zeiss; Oberkochen, Germany) after cryosectioning the skin (16 µm-thick sections) with a microtome (CM1860UV, Leica; Wetzlar, Germany).

### 2.6. Oral Sensitization and Antigen Challenge with Whey Protein

The whey protein diet, AIN-93G-W, in which the milk casein in AIN-93G was substituted with whey protein (WPC392), was custom prepared by Oriental Yeast (Tokyo, Japan). For oral sensitization, 5-week-old mice received the whey protein diet AIN-93G-W for 2 weeks. After a 4-week interval on a CE-2 diet (soy protein-based diet), the mice again received AIN-93G-W for 2 weeks and then were maintained on a CE-2 diet for the remainder of the experiment [15,16]. One week after the final whey feed, mice were challenged orally with 60 mg of whey protein in PBS (0.3 mL) [17]. At 18 h after the antigen challenge, blood was collected from a tail vein. Standard sera for use as a standard in enzyme-linked immunosorbent assays (ELISAs) were obtained from standard model mice maintained on an AIN-93G-W diet for the first 8 weeks, then on a CE-2 diet.

The acute skin response was checked by measuring the ear thickness with a digital micrometer (Mitutoyo; Kanagawa, Japan), 5 min after 10 µg of WPC392 in PBS solution (200 mg/mL) was spread on the backside of the external ears.

### 2.7. Transcutaneous Immunotherapy (TCIT) with S/O Nanodispersion

Gauze patches (0.5 × 1.0 cm^2^) containing 25 μL of the BLG formula (25 μg BLG) were administered over 24 h on the intact ears of whey allergy model mice. Surgical adhesive tapes were used to fix and cover the patches. The BLG patches were administered a total of three times at an interval of once per week. As a control, BLG in a PBS solution was administered by subcutaneous injection following the same schedule as used for TCIT. One week after the final BLG administration, the mice were subjected to an oral antigen challenge (60 mg of whey protein in 0.3 mL of PBS) followed by blood collection. The mice were sacrificed one week after the oral challenge, and the spleens were harvested for cytokine analysis. Each group consisted of six mice.

### 2.8. Measurement of Serum IgE, IgG1, and IgG2a

Serum total IgE levels were determined with an IgE Mouse Uncoated ELISA Kit (Thermo Fisher Scientific; Waltham, MA, USA) used in accordance with the manufacturer’s instructions. Antigen-specific IgG1 and IgG2a levels were also measured by ELISA. Briefly, 96-well Nunc MaxiSorp microplates were coated overnight with 0.5 mg of BLG at 4 °C. The next morning, the plates were washed with PBS-T (0.05% Tween-20 in PBS) and blocked with 2% bovine serum albumin in PBS (200 µl/well) for 2 h at 37 °C, then washed again. The plates were then incubated with serial dilutions of serum samples (50 μL per sample) for 2 h at 37 °C before being washed and incubated with horseradish peroxidase-conjugated rabbit anti-mouse IgG1 or IgG2a antibodies (1:20,000 dilution) for 2 h at 37 °C. After a final wash, the colour was developed with 3,3′,5,5′-tetramethylbenzidine for 30 min at 37 °C, then stopped with 1 M sulfuric acid. The optical densities (ODs) at 450 nm and 570 nm were measured with a microplate reader (Synergy HTX; BioTek, Tokyo, Japan), and the difference in absorbance between the two ODs was used in quantification. Serum from a standard allergy model mouse was used as the calibration standard in the ELISA determination of BLG-specific IgG1 and IgG2a. The BLG-specific IgG1 and IgG2a levels in the standard serum were arbitrarily assigned as being 10,000 relative units (RU)/mL. The data are expressed as the mean and the standard deviation of *n* = 6.

### 2.9. Cytokine Measurement

Mixtures of the spleen cells collected from six mice per group (2 × 10^6^ cells/well) were incubated in a 96-well plate with 100 μg of whey protein (5% CO_2_, 37 °C). After 24 h, the cells were collected, and the cytokine profile in the culture supernatant was measured using ELISA kits from Thermo Fisher Scientific (Waltham, MA, USA). The data shown below are the mean plus the standard deviation of the triplicate tests.

### 2.10. Statistical Tests

Significant differences were determined using a Tukey’s multiple comparison test following a one-way analysis of variance, conducted with Graph Pad Prism 6. The level of significance was set at *p* < 0.05.

## 3. Results

### 3.1. Characteristics of the S/O Nanodispersions

**Figure 1:**
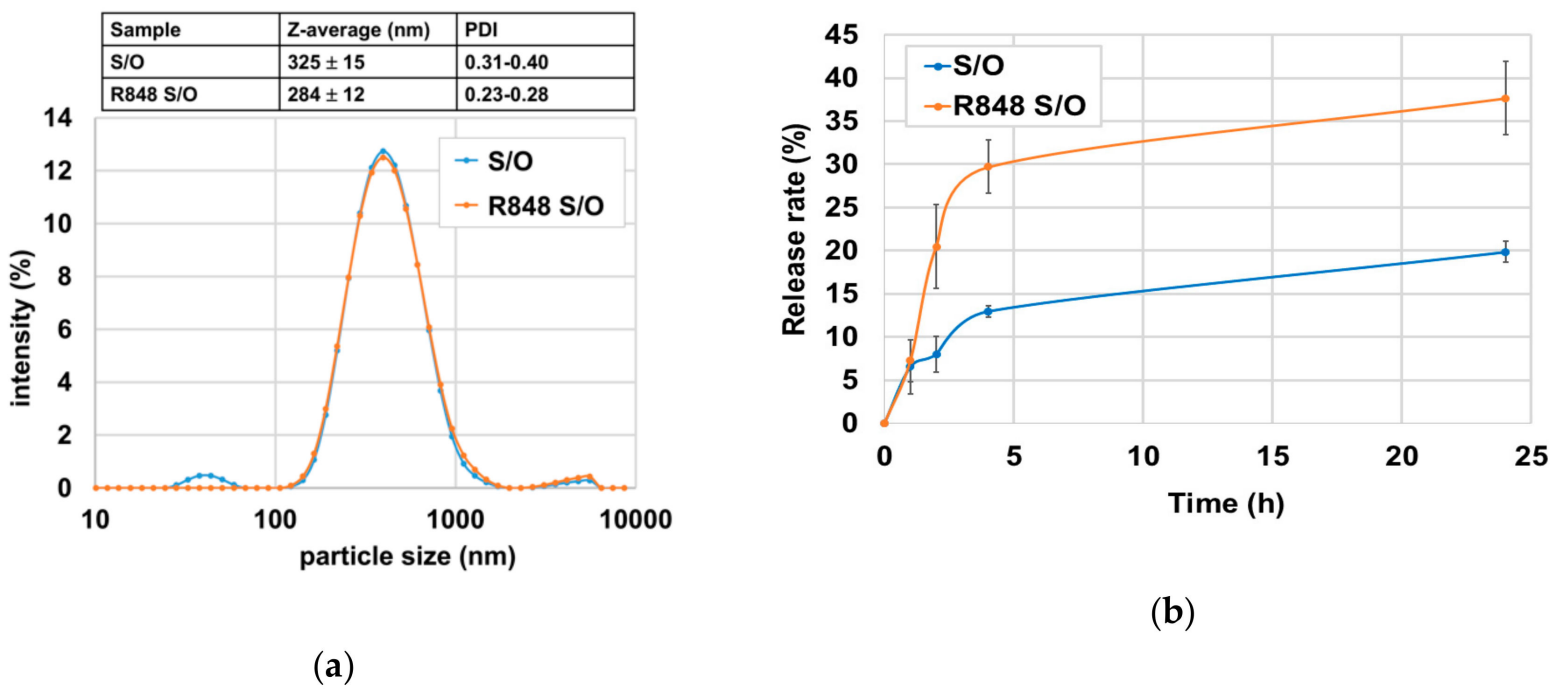
Characterization of the solid-in-oil (S/O) nanodispersions with and without R-848. (**a**) Particle size distribution; (**b**) β-lactoglobulin (BLG) release test.

The S/O nanodispersions contained nanosized particles of hydrophilic molecules in oil vehicles. The particle sizes and the polydispersity index in these S/O nanodispersions were measured by dynamic light scattering. As shown in Figure 1a, the BLG molecules were uniformly dispersed in the S/O nanodispersion. The mean particle size was around 300 nm, and the particle size was not affected by the presence of R-848 in the IPM vehicle. Furthermore, no change in the polydispersity index was detected following the addition of R-848. However, the release efficiency of the BLG molecules was slightly increased by the presence of R-848 (Figure 1b).

### 3.2. In Vivo Skin Permeation

**Figure 2:**
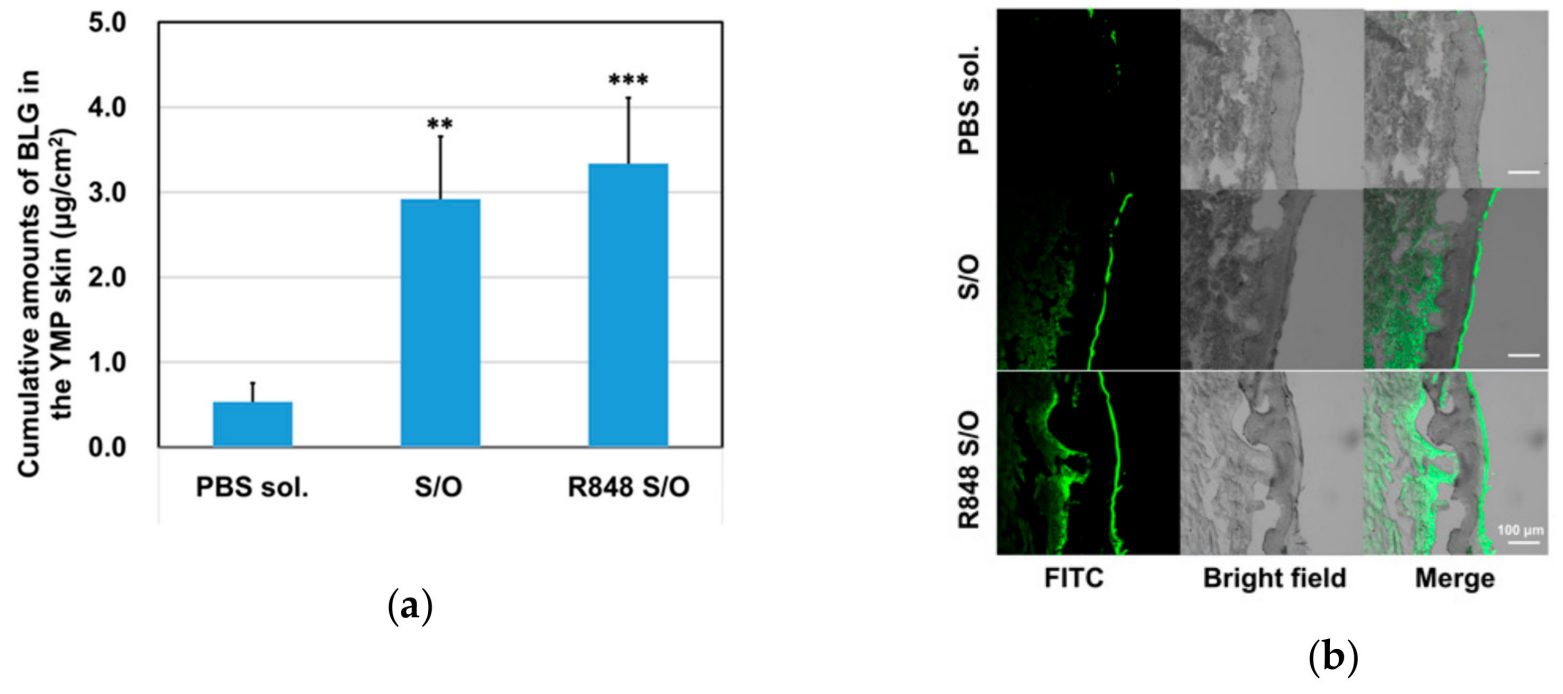
In vitro skin permeation in Yucatan micropig (YMP) skin. (**a**) Cumulative amounts of Fluoroscein 5-isothiocyanate (FITC)-labelled BLG in the solid-in-oil nanodispersion (S/O) and the S/O with R-848 (R-848 S/O) that permeated through the YMP skin after 24 h of incubation. *p < 0.05, **p < 0.01, ***p < 0.001 compared with the PBS solution (PBS sol.).; (**b**) Representative fluorescent microscopy images of the YMP skin sections after 24 h of BLG administration.

The skin permeability of S/O nanodispersions carrying FITC-BLG was examined using YMP skins set onto Franz diffusion cells. The amount of FITC-BLG that permeated through the skin was more than five-fold higher for the S/O nanodispersion compared with that for the PBS solution (Figure 2a). Permeations of FITC-BLG into the dermis were also observed in the fluorescent microscopy images of the sectioned skins, following the application of the S/O nanodispersion technique (Figure 2b).

### 3.3. Humoral and Cellular Immune Responses and Physical Symptoms in Model Mice

**Figure 3:**
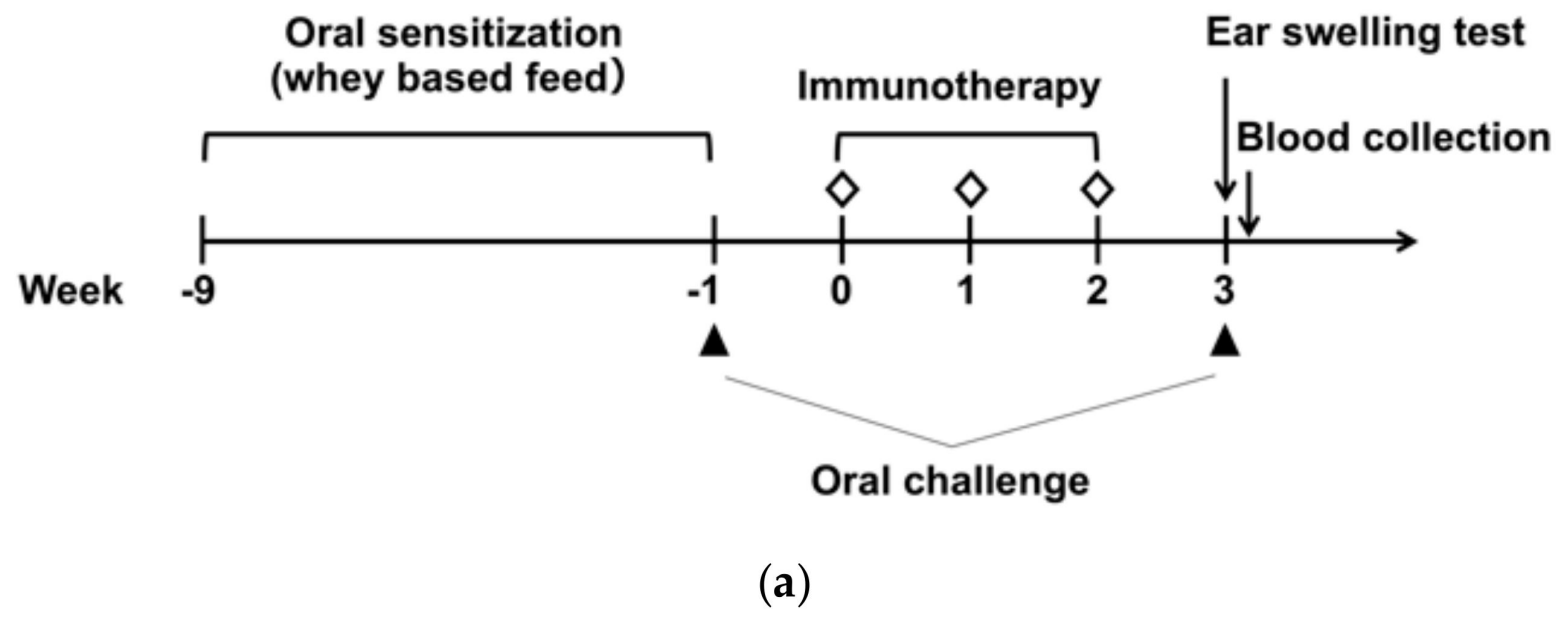

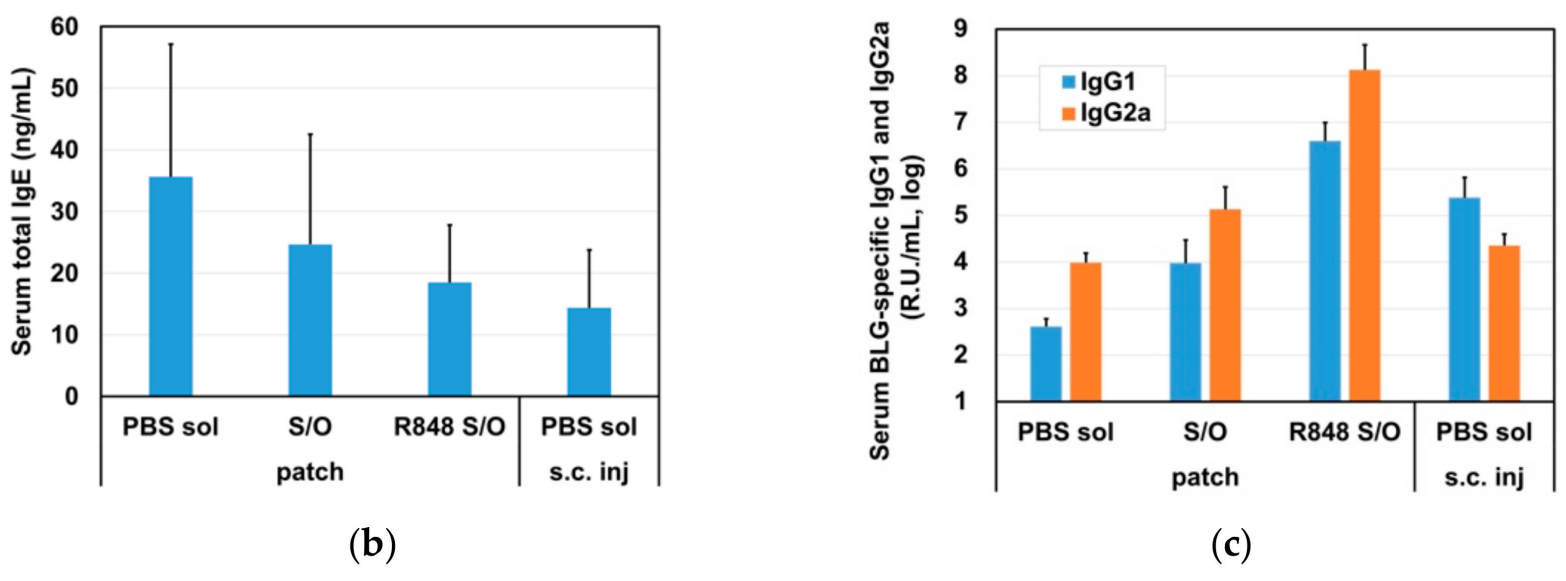
Serum antibody responses after antigen immunotherapy. (**a**) Schematic of the transcutaneous immunotherapy (TCIT) schedule. Mice were treated with a patch containing S/O nanoparticles carrying BLG without (S/O patch) or with (R848 S/O patch) R-848 in an IPM vehicle or a PBS-based solution of BLG (PBS sol. patch) or were subcutaneously injected with a PBS-based solution of BLG (PBS sol. s.c. inj). (**b**,**c**) The levels of total IgE (**b**) and BLG-specific IgG1 and IgG2a (**c**) in sera from mice treated as described in (**a**) were determined by enzyme-linked immunosorbent assay (ELISA). BLG dissolved in PBS (PBS sol.) was administered as a negative control. Data are shown as the mean + SD (*n* = 6; no significant differences were observed).

TCIT was performed following the schedule shown in Figure 3a. Mice were treated with a patch containing S/O nanoparticles carrying BLG without (S/O patch) or with (R848 S/O patch) R-848 in an IPM vehicle or a PBS-based solution of BLG (PBS sol. patch) or were subcutaneously injected with a PBS-based solution of BLG (PBS sol. s.c. inj). After BLG immunotherapy via subcutaneous injection or via the S/O nanodispersion system, the serum total IgE levels trended lower (Figure 3b) compared with those in mice patched with a BLG aqueous solution; however, none of these differences achieved statistical significance. Inversely to the relatively low total IgE levels, the BLG-specific IgG subclass levels trended higher in the sera of mice treated with the S/O nanodispersion system (Figure 3c). Specifically, the serum levels of BLG-specific IgG2a trended higher in the S/O nanodispersion group, but not in the subcutaneous BLG injection group, compared with naïve mice. Although the differences did not reach statistical significance, the levels of secreted BLG-specific IgG2a trended even higher following the transcutaneous administration of R-848 with BLG in the S/O nanodispersion system, whereas a similar increase was not observed in mice treated with a subcutaneous injection of BLG.

**Figure 4:**
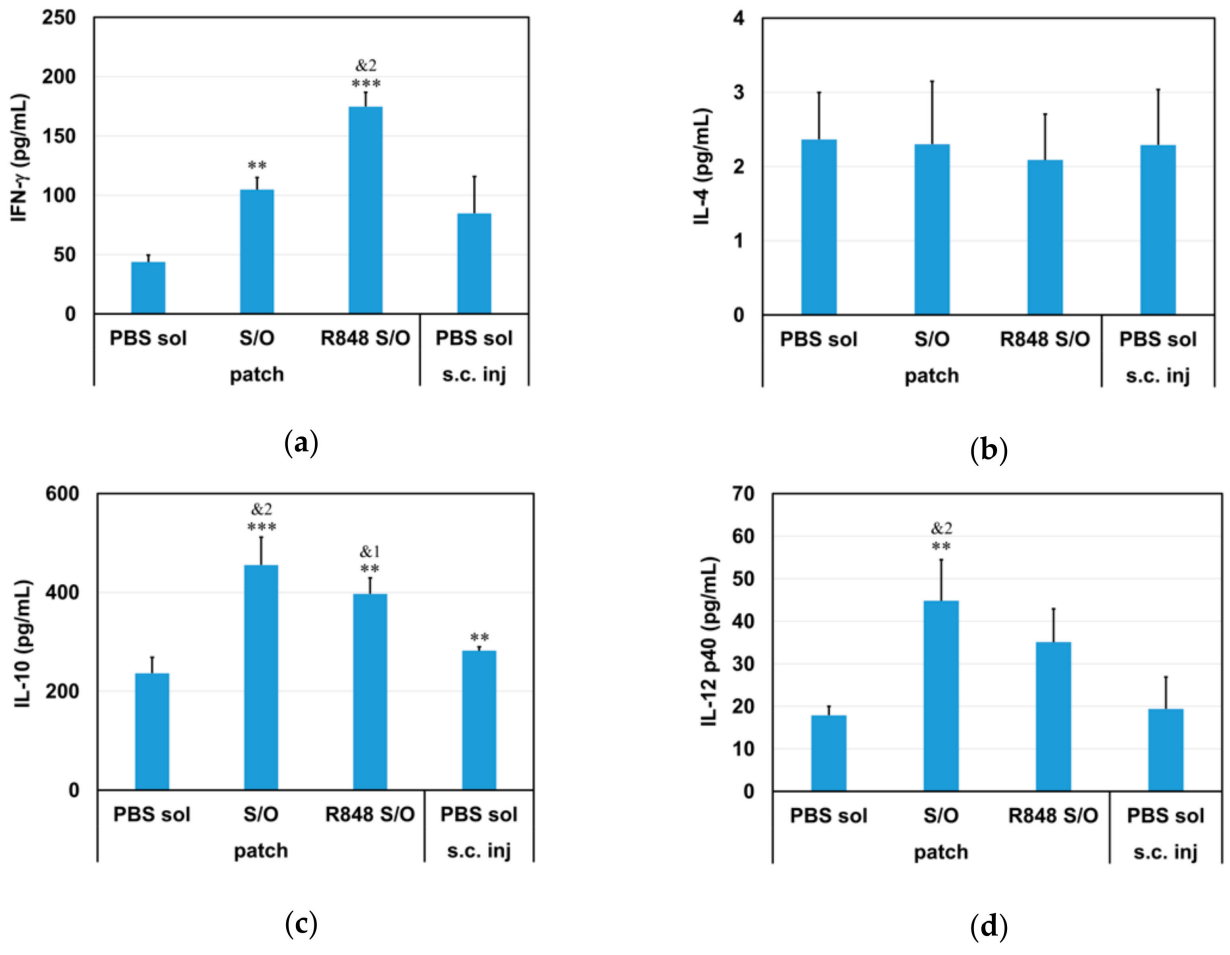

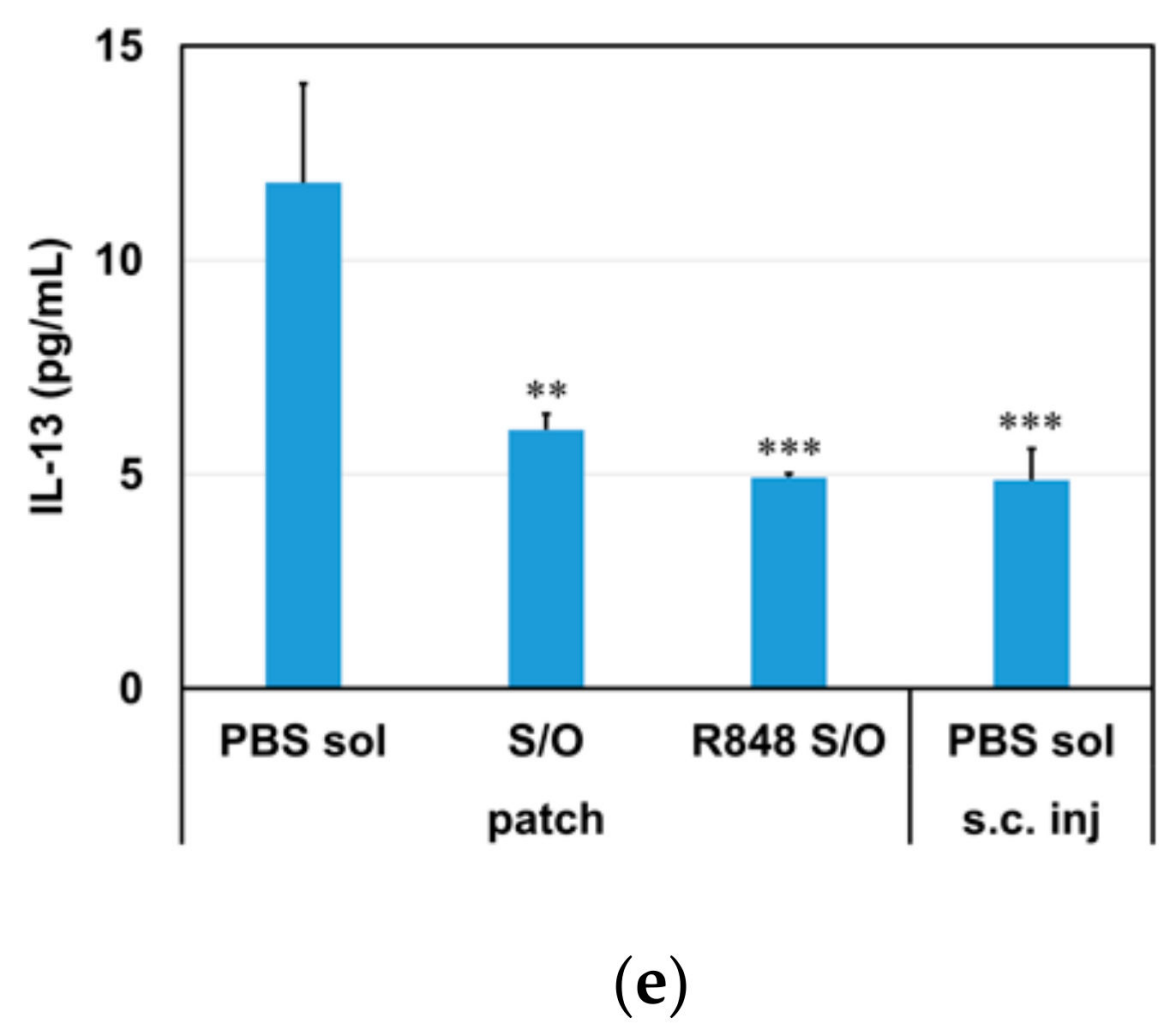
Cytokine levels after splenocyte stimulation with whey protein. (**a–e**) Levels of IFN-γ (**a**), IL-4 (**b**), IL-10 (**c**), IL-12 p40 (**d**), and IL-13 (**e**) in the supernatant of splenocytes from mice treated as described in Figure 3a following stimulation with whey protein. The cytokine concentrations in the cell cultures 24 h after stimulation with antigen were determined using sandwich ELISA kits. Data are shown as the mean + SD (*n* = 6). Statistical analysis was performed using an ANOVA and Tukey’s post-hoc test for multiple comparisons. *, *p* < 0.05; **, *p* < 0.01; and ***, *p* < 0.001 compared with the BLG dissolved in PBS (PBS sol); &1, *p* < 0.05 and &2, *p* < 0.01 compared with the subcutaneously injected BLG in PBS solution (PBS sol. s.c. inj).

The profile of cytokines secreted by antigen-stimulated cultured splenocytes from the treated mice were examined (Figure 4). Although the IL-4 levels were low overall in the C3H mice used here, and did not differ between the four groups (S/O patch, R848 S/O patch, PBS sol. patch, and PBS sol. s.c. inj), the IL-13 levels were lower in splenocytes from mice administered BLG via the S/O technology (S/O patch or R848 S/O patch) or via subcutaneous injection (PBS sol. s.c. inj) compared with those from the PBS sol. patch mice. In the subcutaneous injection group (PBS sol. s.c. inj), the IFN-γ level was relatively low compared with the higher levels in the S/O groups (S/O patch or R848 patch). Additionally, the IL-10 and IL-12 p40 levels were also relatively higher in the S/O groups.

**Figure 5:**
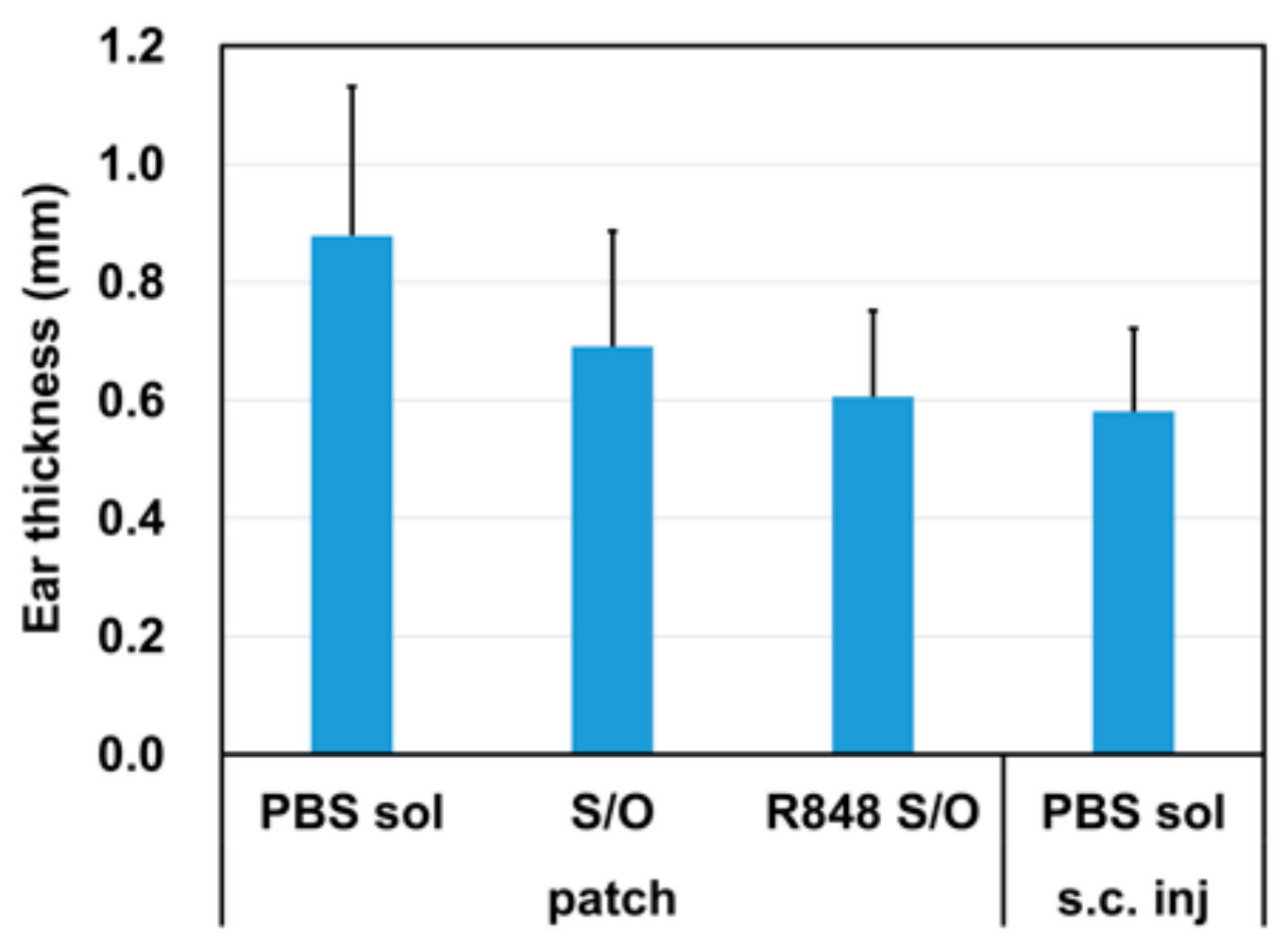
Acute skin reactions. The ear thicknesses of mice treated as described in Figure 3a were measured 5 min after skin contact with whey protein.

The acute skin responses in the model mice were measured within 5 min of contact with the antigen. The amount of observed ear swelling trended highest in the mouse group treated with patches carrying a PBS-based solution of BLG (Figure 5) compared with the mouse groups treated with BLG administered via the S/O system or the subcutaneous injection method.

## 4. Discussion

CMA frequently emerges in infants, most of whom outgrow the allergy by the age of 3–5 years; however, it is a severe concern for the individuals in whom this allergy persists given the risks of accompanying atopic disorders, asthma, anaphylaxis, and other allergen-related chronic or acute symptoms [1]. Immunotherapy is considered to be the only curative treatment method for allergies [4]. This study used a mouse model to investigate the utility of TCIT via the S/O nanodispersion system for the treatment of CMA. The S/O nanodispersion system consists of nanoparticles of protein coated with surfactant molecules that have a low hydrophilic-lipophilic balance (HLB) value (<2) and of an oil vehicle that is compatible with the lipids in the surface layer of the skin [18]. As the enhancement of the protein/peptide permeation through the skin of this approach is significant when compared with that of an aqueous solution of the same protein/peptide, the S/O nanodispersion system has been successfully applied to vaccine delivery through the skin [7].

The S/O nanodispersion containing BLG was successfully prepared using sucrose laurate L-195, for which the HLB value is approximately 1. In a preliminary study, we examined surfactant protein ratios of 1:25, 1:50, and 1:100, and we found that the nanodispersion was unstable when the ratio was 1:25. As higher surfactant concentrations were prone to inhibiting the protein permeation through the skin [19,20,21], we employed a surfactant:protein ratio of 1:50 for preparing the S/O nanodispersions in this study. The mean particle size of the S/O dispersions used here was around 300 nm. However, the release efficiency was slightly increased by the presence of R-848, suggesting an interaction between R-848 and the nanoparticles. 

The results of the in vitro skin-permeation test indicate that the S/O system successfully facilitated the permeation of the protein-drug through the YMP skin, which was used as a human skin model. Previous work found that the toll-like receptor 7 agonist R-848 suppresses allergic asthma when transcutaneously administered in a mouse model of birch pollen allergy [22]. Thus, we examined the additional use of R-848 in this model for CMA immunotherapy. The addition of R-848 did not alter the particle sizes or polydispersion index of the S/O dispersions. Histological observations of YMP skin sections treated as described above indicated that FITC-BLG permeated into the dermis when it was encapsulated in S/O nanoparticles, and that the skin permeation efficiency was not affected by the presence of R-848. More importantly, BLG was observed in the whole SC, suggesting that the SC may act as a reservoir that stores the S/O nanoparticles and gradually releases the proteins.

S/O nanodispersion patches were administered to the CMA model mice once per week for 3 weeks. As the CMA model mice showed ear swelling symptoms in a previous report [23], we measured the ear thickness of these mice at 5 min after skin contact with the antigen. The amount of ear swelling trended highest in the group treated with BLG in a PBS-based solution compared with the amounts in the groups treated with S/O nanodispersions or s.c. injections, which had minimal allergic skin responses. Moreover, the amount of ear swelling in the group treated with an S/O nanodispersion that also contained R-848 trended lowest, indicating that this immunomodulator may improve the effectiveness of TCIT.

Following the administration of BLG via subcutaneous injection, the total IgE level trended lower, and the IgG1 antibody level trended higher, with no apparent trends in the IgG2a level, as compared with the PBS solution groups. In contrast, while the total IgE level also trended slightly lower in the S/O nanodispersion groups, both the IgG1 and IgG2a levels in these groups trended higher compared with the other groups. This trend agrees with the reported induction of IgG subclass antibodies in OIT-treated patients [24]. IgG antibody is supposed to inhibit IgE-mediated anaphylaxis through the interception of antigen molecules and the cross-linking of FcεRI-FcγRIIb [25]. We also observed changes in the cytokine levels after treatment. Compared with the groups treated with S/O dispersions, the cytokine production levels were generally lower in the groups treated with PBS-based solutions of BLG, except for IL-13, regardless of whether it was administered in a patch or via a subcutaneous injection. 

The S/O dispersion groups had relatively higher levels of IFN-γ and relatively lower levels of IL-13, indicating that the normal T helper type 2 (Th2-type) immune response was skewed toward Th1-type immunity after S/O dispersion patch administration. A reduction in IL-13 levels, in addition to reductions in the IL-4 and IL-5 levels, was similarly observed after OIT but not after EPIT in a previous study [24]. Thus, the immunotherapeutic mechanism of TCIT with a S/O nanodispersion appears to be more similar to OIT than to EPIT. The difference may be a consequence of the region in the skin to which the antigens are delivered; they reach the epidermis and dermis in TCIT, whereas they reach only the epidermis in EPIT. 

The observed higher level of IL-12 p40 in the S/O groups may indicate an activation of Langerhans cells [26]. Regulatory T cell-derived IL-10 was previously reported to be responsible for the immunosuppression of allergy-related cells [27,28]. Hence, the induction of IL-10 in our results may indicate the amelioration of allergic immune reactions. Notably, an amelioration of the physical allergic response was also observed. The ear swelling response tended to be alleviated by the BLG administration using the S/O system. Other promising approaches to deliver vaccines for CMA immunotherapy have recently been developed, including the oral administration of PLGA nanoparticles [29]. However, transcutaneous administration may still be more convenient for patients who are young infants or who have gastrointestinal disorders.

R-848 is an agonist that binds to toll-like receptor 7/8 and has an inhibitory effect on Th2-type immunity via initiating the Th1-type immune response [30]. As allergies are related to a Th2 cell-driven immune response, R-848 is beneficial for the alleviation of allergic symptoms including allergic rhinitis [31], asthma [22], and inflammation [32]. Additionally, unlike the occlusive patch system, the S/O system is able to efficiently deliver lyophilic compounds through the SC due to the oil-vehicle. The skewing of the immune response toward Th1-type immunity was more pronounced when R-848 was co-administrated with the S/O nanodispersion, indicating that this immunomodulator has a synergistic effect with the antigen in TCIT for this model.

## 5. Conclusions

Here, we applied an S/O nanodispersion system to TCIT for CMA in a mouse model of allergy. The use of this system enhanced the antigen permeation through the YMP skin more than five-fold compared with the use of a simple solution of the same antigen in PBS. After the treatment of whey-sensitized mice with S/O nanodispersions carrying the antigen BLG, the level of total IgE trended lower and the levels of BLG-specific IgG subclasses trended higher compared with similar model mice treated with BLG in a PBS solution. Moreover, the profile of cytokines secreted by splenocytes from these mice indicates the skewing of the immune reaction toward Th1-type immunity after S/O treatment. The amount of ear swelling also trended lower following antigen immunotherapy using the S/O technology. The addition of the immunomodulator R-848 into the S/O system reinforced the Th1-type immune response in the mouse model. The S/O nanodispersion technology is simple enough that self-medication would be possible; therefore, it is a promising approach for transcutaneous antigen immunotherapy to treat CMA.
